# Astrocytes in Tauopathies

**DOI:** 10.3389/fneur.2020.572850

**Published:** 2020-09-24

**Authors:** Matthew J. Reid, Paula Beltran-Lobo, Louisa Johnson, Beatriz Gomez Perez-Nievas, Wendy Noble

**Affiliations:** Department of Basic and Clinical Neuroscience, Institute of Psychiatry, Psychology and Neuroscience, King's College London, London, United Kingdom

**Keywords:** tau, astrocyte, tauopathy, prion-like propagation, Alzheimer's disease, glia

## Abstract

Tauopathies are a group of neurodegenerative diseases characterized by the progressive accumulation across the brain of hyperphosphorylated aggregates of the microtubule-associated protein tau that vary in isoform composition, structural conformation and localization. Tau aggregates are most commonly deposited within neurons but can show differential association with astrocytes, depending on the disease. Astrocytes, the most abundant neural cells in the brain, play a major role in synapse and neuronal function, and are a key component of the glymphatic system and blood brain barrier. However, their contribution to tauopathy progression is not fully understood. Here we present a brief overview of the association of tau with astrocytes in tauopathies. We discuss findings that support a role for astrocytes in the uptake and spread of pathological tau, and we describe how alterations to astrocyte phenotype in tauopathies may cause functional alterations that impedes their ability to support neurons and/or cause neurotoxicity. The research reviewed here further highlights the importance of considering non-neuronal cells in neurodegeneration and suggests that astrocyte-directed targets that may have utility for therapeutic intervention in tauopathies.

## Introduction

Tauopathies are a heterogeneous group of neurodegenerative diseases in which the deposition of hyperphosphorylated tau aggregates in affected brain regions accompanies synapse and neuron loss (1, 2). Primary tauopathies exhibit tau aggregates as the predominant pathological hallmark and include a diverse family of frontal-temporal lobar dementia (FTLD) subtypes referred to as FTLD-tau, and progressive supranuclear palsy (PSP) and Pick's disease (PiD). Alzheimer's disease (AD) is considered a secondary tauopathy owing to the presence of extracellular amyloid-beta (Aß) plaques, and is the most common cause of dementia (1).

Tau proteins undergo several post-translational and other modifications in disease (2). Modified forms of tau spreads from the original site of deposition to anatomically connected regions by a “prion-like” mechanism, whereby tau proteopathic seeds passively recruit tau monomers (3). The mechanisms underlying tau release, uptake and spread are not fully understood. It has long been acknowledged that in some tauopathies astrocytes accumulate tau leading to characteristic disease neuropathology. Accumulating evidence now suggests that astrocytes may actively participate in tau spread and/or clearance mechanisms by actively internalizing tau. This review summarizes the association of tau with astrocytes in tauopathies, and discusses the evidence implicating astrocytes in tau spread, as well as the impact of tauopathy brain environments on physiological astrocytic functions.

## Tau Protein

Human tau is encoded by the *MAPT* gene on chromosome 17 which comprises 16 exons. Exons 2, 3, and 10 undergo alternative splicing to produce the six main tau isoforms present in the adult human central nervous system (CNS) (4). Alternative splicing of exon 10 gives rise to tau isoforms containing either three or four microtubule binding repeats (referred to as 3R or 4R tau) in the C-terminal region, and alternative splicing of exons 2 and 3 produces tau proteins with zero, one or two inserts in the N-terminal tail (0N, 1N, or 2N tau, respectively). A conserved proline-rich domain is found between these two spliced regions and is known to be important for tau interactions with other proteins, including actin (5). Tau isoforms are developmentally regulated; the shortest 0N3R isoform is expressed in the fetal brain whereas in the adult human brain 3R and 4R isoforms are equally represented (6). Tau has a number of key functions, the most recognized of which is stabilizing microtubules in the axons of neurons, however tau roles in other important physiological functions such as axonal transport, DNA protection, cell signaling at the membrane, and synaptic vesicle release, have been described (2, 7). Tau is primarily expressed in neurons (8), but is known to be expressed to a lesser extent in glial cells (9–12).

Monomeric tau is water soluble and resists aggregation (7). In tauopathies, tau undergoes extensive post-translational and other modifications including, but not limited to, phosphorylation, acetylation, nitration, SUMOylation, glycosylation, ubiquitination, cleavage, and aggregation (2). The best studied of these is phosphorylation. There are 85 potential phosphorylation sites in 2N4R tau (13) and increased phosphorylation of tau, alongside other tau modifications, can reduce tau affinity for microtubules, increase cytoplasmic tau concentrations and promote tau oligomerisation and aggregation (2). Differential extents of tau modifications lead to the accumulation of heterogeneous pools of modified tau between, and within, different tauopathies. Recently, Dujardin et al. (14) found variations in the relative abundance of soluble, oligomeric and seed-competent species of hyperphosphorylated tau in tauopathy brain. Specific post-translational modifications were found to influence tau seeding capacity, and tau seeding potential strongly correlated with the rate of clinical symptoms/disease progression.

The isoform composition of tau aggregates, as well as the structure of tau filaments, also differs between tauopathies. In AD, both paired helical and straight filaments contain identical protofilament cores comprising residues 306–378 that define the aggregatory seed/core (15). This structure differs from the folds of tau filaments observed in Pick's disease (16) and tau filaments of chronic traumatic encephalopathy (CTE) have a unique hydrophobic core (17). A novel fold in corticobasal degeneration (CBD) tau has now also been discovered (18). These features may be important for the tau lesions that arise in different tauopathies (Table 1).

**Table 1:** Overview of the main clinical, genetic, molecular, and pathological features of tauopathies, including description of astrocyte abnormalities.

**Disease**	**PiD**	**PSP**	**CBD**	**AGD**	**GGT**	**ARTAG**	**AD**	**PART**	**CTE**
Common clinical symptoms	Aphasia, several behavioral changes including and personality changes, cognitive changes at later stages of disease.	Balance and motor deficits, dysphagia and aphagia.	Motor problems (often one-sided), aphagia, dysphagia.	Amnestic mild cognitive impairment often accompanied by neuropsychiatric symptoms.	Behavioral changes, mood swings, short-term memory loss.	Often no cognitive impairment or dementia related symptoms. Focal pathology may correlate with specific deficits, especially in the presence of co-pathology.	Dementia; progressive episodic memory deficits; navigational and multi-tasking difficulties; diverse behavioral and personality changes.	Associated with cognitive impairment and mild AD-like symptoms.	Behavioral changes, mood swings, short-term memory loss.
MAPT cause/risk	Mostly sporadic; MAPT mutations (exon 9, 10, 11, 12, 13 and intron 9, 10).	Mostly sporadic, H1/H1c MAPT haplotype increases risk; MAPT mutations (exon 1, 10, and intron 10);	Mostly sporadic; H1 MAPT haplotype increases risk; MAPT mutations (exon 10, 13 & intron 10);	H1 MAPT haplotype may increase risk; MAPT mutations (exon 10)	H1 MAPT haplotype; MAPT mutations (exons 1, 10, 11, intron 10).	*Depending on sub-type and classification*	Mostly sporadic; APP, PSEN1, PSEN2; No MAPT mutations	*Depending on sub-type and classification*	*Unknown* (external causes)
Primary tau isoforms that accumulate in lesions	3R	4R	4R	4R	4R	4R	3R & 4R	3R & 4R	3R & 4R
Affected brain regions	Frontal and temporal cortices.	Precentral cortex, subcortex (globus pallidus, substantia nigra, pontine nuclei, subthalamic nuclei).	Frontal and temporal cortices.	Medial temporal lobe.	Frontal, precentral and/or temporal cortices.	Gray and/or white matter, perivascular, subpial, subependymal.	Entorhinal cortex and hippocampus, spreading to most regions except the cerebellum.	Entorhinal cortex, hippocampus.	Begins focally at depths of cerebral sulci, spreads widely to frontal temporal lobes.
Hallmark astrocytic tau pathology	Ramified	Tufted	Astrocytic plaques	Thorn-shaped & granular fuzzy/bush-like	Globular inclusions	Thorn-shaped & granular fuzzy	*None*	*None*	Astrocytic tangles and some thorn-shaped astrocytes.
Cellular localization of astrocytic tau inclusions	Asymmetric 3R (predominant) or 4R tau inclusions in cell bodies & proximal processes.	Symmetric 4R tau inclusions in proximal processes.	4R tau in distal processes and end feet; thread-like processes are also common.	4R tau inclusions and diffuse staining in cell bodies & proximal-distal processes.	4R globular tau in cell bodies & proximal processes.	4R tau inclusions and diffuse staining in cell bodies & proximal processes.	*n/a*	*n/a*	Irregular p-tau lesions (around small vessels).
References	Forrest et al. (19, 20); Dickson et al. (21); Dickson (22); Josephs et al. (23); Ferrer et al. (24).	Forrest et al. (19, 20); Cairns et al. (25); Kovacs and Budka (26).	Forrest et al. (19, 20); Dickson et al. (21); Ling et al. (27).	Forrest et al. (19, 20); Botez et al. (28); Rodriguez and Grinberg (29); Saito et al. (30).	Forrest et al. (19, 20); Ahmed et al. (31).	Forrest et al. (19, 20); Kovacs et al. (32); Kovacs (33, 34); Kovacs et al. (35);	Guerreiro et al. (36); Braak and Braak (37); Braak et al. (38); Lane et al. (39).	Forrest et al. (19, 20); Crary et al. (40); Jellinger et al. (41).	Forrest et al. (19, 20); Stein et al. (42); McKee et al. (43, 44).

*PiD, Pick's disease; PSP, progressive supranuclear palsy; CBD, corticobasal degeneration; AGD, argyrophilic grain disease; GGT, globular glial tauopathy; ARTAG, age-related tau astrogliopathy; AD, Alzheimer's disease; PART, primary age-related tauopathy; CTE, chronic traumatic encephalopathy; 3R, 3-repeat tau; 4R, 4-repeat tau*.

## Astrocytes in Health and Disease

Astrocytes are organized into distinct domains, and each astrocyte can connect with thousands of neurons, allowing them to coordinate synaptic activity in the CNS (45, 46). Astrocytes were long considered as supporting cells in the brain, providing metabolic and nutritional support for neurons. However, astrocytes are critical for neuronal function due to their ability to sense changes in neuronal activity through their complement of cell surface receptors, and to modulate neuronal activity by releasing gliotransmitters and gliomodulators, as well as controlling the availability of glutamate, GABA, and energy substrates (45, 47, 48). Hence, astrocytes are now known to be actively involved in synaptic transmission (49), neural circuit maintenance (50) and long-term potentiation (51). In addition, astrocytic end-feet are a structural component of the blood-brain barrier (BBB), and together with endothelial cells and pericytes have a central role in the regulation of blood flow (52). Furthermore, astrocyte end-feet are crucial for the glymphatic system of the brain, a perivascular network that allows for exchange of interstitial and cerebrospinal fluid (CSF), providing a route for clearance of molecules and proteins including Aβ (53, 54).

In neurodegenerative disease brain, astrocytes undergo pathological changes in responses to changes in the local brain environment that precede neuronal loss (55). These morphologically and functionally modified astrocytes are often termed “reactive.” Reactive astrocytes show considerable heterogeneity related to their localization in the brain and the severity and length of injury/insult to their local environment (56). Reactive astrocytes are traditionally characterized by increased levels of glial fibrillary acidic protein (GFAP), which allows cytoskeletal and morphological arrangements as astrocytes alter their function (57, 58). The accumulation of GFAP-immunopositive astrocytes is common in neurodegenerative diseases. For example, reactive astrocytes are often found surrounding plaques in AD (59, 60). Indeed, levels of GFAP-reactive astrocytes are closely associated with dementia in AD (61). While increased GFAP is also found in aged brain (62), new evidence suggests that there are subgroups of astrocytes, with varying levels of GFAP expression, that distinguish aging from AD, at least in mice (63). Alterations in GFAP expression have also been noted in primary tauopathies including PSP, PiD and corticobasal degeneration (CBD) (24).

Functional changes in reactive astrocytes are well-documented and include impaired gliotransmitter release (64), alterations in calcium signaling (65), deficient ability to regulate glutamate levels at neuronal synapses and aberrant GABA release (58). In addition, astrocytes are now recognized to contribute to neuroinflammatory responses that accelerate the progression of neurodegenerative diseases (59, 66, 67). For example, reactive astrocytes increase their production and release of pro-inflammatory cytokines, complement components, and reactive oxygen species, alongside downregulating anti-inflammatory, and repair proteins to induce neurotoxicity in diseased environments (59, 68–70). Recent seminal findings proposed that astrocytes respond to their local environment by adopting “A1-neurotoxic” or “A2-neuroprotective” phenotypes (71). Secretion of Il-1α, TNFα, and C1q by microglia in response to damage, induces astrocytes to upregulate their expression of a specific cluster of “A1” genes, lose their trophic and synaptic support for neurons, and induce neuron death (71). Markers of A1 astrocytes are upregulated in AD and other neurodegenerative diseases (71), strongly implicating microglia-astrocyte communications in neurodegeneration. However, it is likely that there is a spectrum of reactive astrocyte states in different brain regions, throughout aging and disease progression (63, 72), similar to dynamic microglial responses in disease (25).

## The Association of Astrocytes With Tauopathy

Tau aggregates accumulate in both neurons and astrocytes in different tauopathies. In AD, tau aggregates containing both 3R and 4R tau deposit as intraneuronal neurofibrillary tangles and there is scant evidence of astrocytic tau inclusions (73). In contrast, astrocytic tau pathology is the defining feature of several FTLD-tau subtypes (Table 1). In PSP, a neuropathological diagnosis criterion is “tufted” astrocytes that show 4R tau aggregates in their proximal processes (26, 74). CBD has extensive clinical overlap with PSP. In CBD, astrocytic plaques containing 4R tau deposits that mark distal and end processes are an exclusive feature in most (19), but not all cases (75). Thread-like tau-positive astrocytic processes are also common in CBD (21, 27). Argyrophilic grain disease (AGD) is a rare tauopathy that is characterized by 4R tau-immunopositive astrocytes, described as thorn-shaped and fuzzy/bush-like, in the medial temporal lobe (19, 28, 30). In contrast, PiD is typically characterized by neuronal 3R tau inclusions, predominantly in granular neurons in the hippocampus, frontal and temporal cortices (22, 23). “Ramified” astrocytes immunopositive for tau have also been reported in PiD, but they are not considered a major pathological hallmark of the disease (21, 24). Several rarer tauopathy subtypes that show 4R tau-immunopositive globular inclusions, predominantly in oligodendrocytes, and more rarely in the cytoplasm, and proximal processes of astrocytes, are collectively termed globular glial tauopathy (GGT) (31).

A spectrum of FTLD-tau subtypes that accumulate both 3R and 3R tau in neurofibrillary tangles (NFTs), typically occurring in cognitively normal aged individuals, is referred as primary age-related tauopathy (PART) (40, 41). Depending on the co-occurrence of Aß pathology, PART can be histologically classified as “definite PART” in the absence of Aß deposits, or “possible PART” when a limited number of Aß deposits are present (40). Although the neuropathological characteristics of PART can overlap with other tauopathies, particularly AD, PART shows a lower threshold of amyloid load, and appears to have a more limited impact on cognition (40, 76). Tau pathology in PART is predominantly neuronal and found in the CA2 hippocampal subfield, with little evidence of astrocytic tau deposits (40, 77). In contrast, age-related tau astrogliopathy (ARTAG) describes a spectrum of abnormal tau pathology, predominantly in the aged brain, that is characterized by thorn-shaped and granular or fuzzy astrocytes containing phosphorylated tau (32, 33). ARTAG can present alongside more typical tau pathology in tauopathies such as CBD (33, 34), but is not always linked with dementia (78). In a recent detailed review, Kovacs (34) describe two distinct distribution patterns of ARTAG. They describe ARTAG as a consequence of repeated mechanical damage (related to CTE), or chronic damage such as blood-brain barrier dysfunction. Furthermore, they propose that the location and type (white vs. gray matter) of ARTAG pathology may result in decompensation of cognitive functions, the rate of which may be influenced by co-existing pathologies (34). It is important to note that the presence of astrocytic tau accumulations in the absence of dementia may suggest that tau-containing astrocytes are not damaging in tau-associated neurodegeneration, or at least in ARTAG, and may internalize tau aggregates as a means of clearing damaging protein species.

Finally, chronic traumatic encephalopathy (CTE) is caused by mild repetitive head injuries. 3R and 4R tau-positive aggregates are common in CTE, however the tau aggregates that accumulate in astrocytes are predominantly 4R and localize in astrocytes near small vessels in the cerebral sulci of the frontal and temporal cortices (42, 43, 79). Thorn-shaped astrocytes are also observed subpial and periventricular regions, an interesting link to ARTAG (34, 44).

## Do Astrocytes Contribute to Tau Pathology Spread?

**Figure 1:**
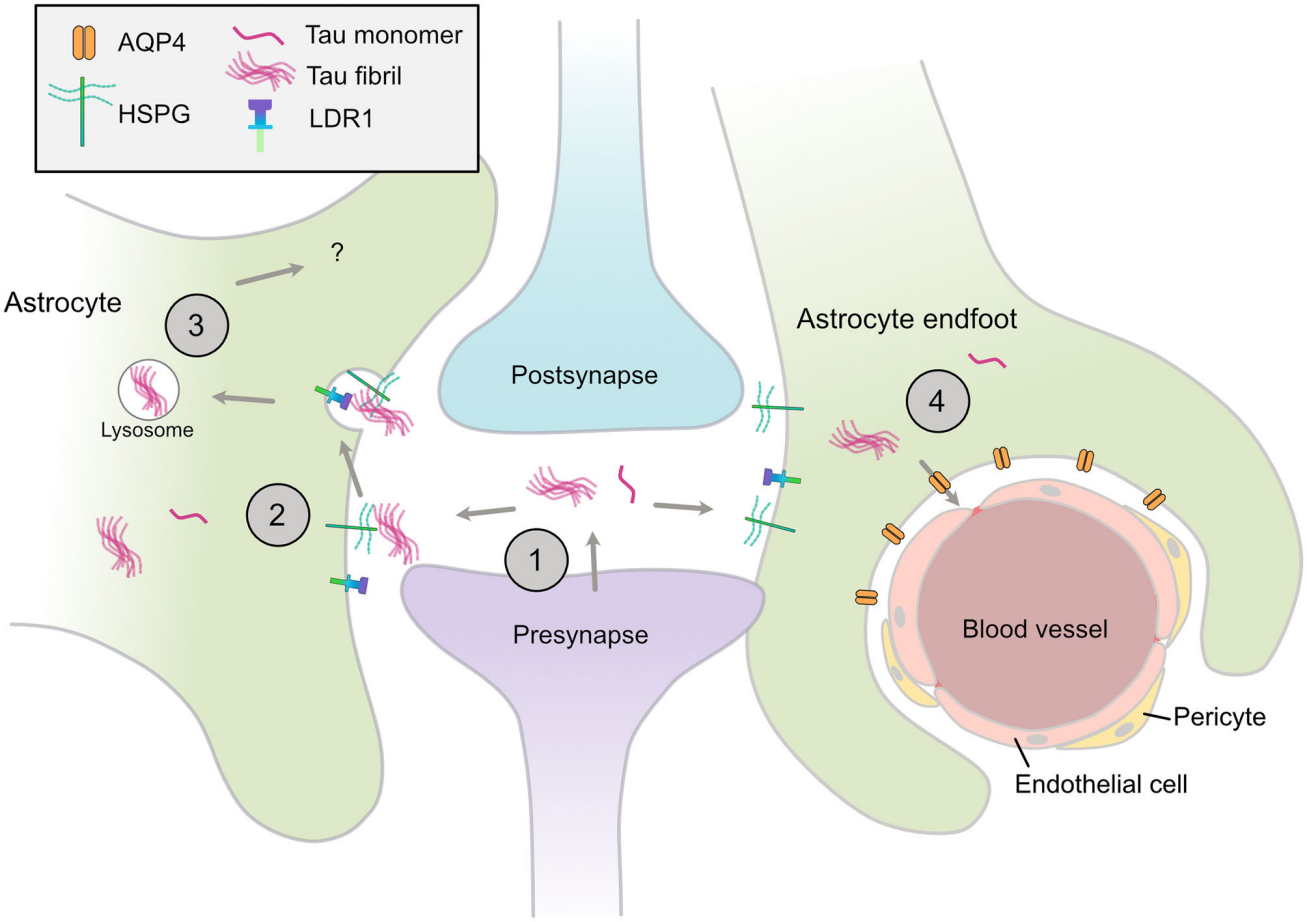
Astrocytic mechanisms that may contribute to spread of tau pathology. (1) Tau monomers and aggregates are released from neurons *via* various mechanisms, including from the pre-synapse, (2) Astrocytes have specific HSPGs and receptors such as LDR1 that may mediate the uptake of tau aggregates, (3) These aggregates may be internalized and processed by various mechanisms, include lysosomal degradation, (4) Disruption of AQP4 in perivascular astrocytic end-feet may contribute to the disrupted tau clearance and the accumulation of tau aggregates in the CNS. HSPG, heparin sulfate proteoglycan; LDR1, low density lipoprotein receptor-related protein 1; AQP4, aquaporin-4.

Neurofibrillary tangles have long been acknowledged to follow a stereotypical temporospatial pattern of spread from the entorhinal cortex as AD progresses (38). Recent evidence indicates that differences in the tau species that deposit in characteristic tau lesions may confer specific neuronal vulnerabilities and/or prion-like spread of tau (14, 80). Mouse models that express wild-type 3R and 4R human tau isoforms in appropriate ratios recapitulate the same cell type vulnerabilities that typify human tauopathies when injected with human tau extracts, including the development of tufted astrocytes in PSP tau-injected mice, and astroglial plaques in CBD tau-injected mice (81). These data raise the possibility that astrocytes actively contribute to the spread of pathological forms of tau, particularly in PSP and CBD. That tau spreads in a prion-like manner trans-synaptically along anatomical connections was elegantly shown in transgenic mice in which mutant human (P301L) FTLD-causing tau expression was restricted to layer II neurons in the entorhinal cortex. Following local tau aggregation, tau “seeds” were found to spread to the hippocampus and onwards as mice aged (82, 83). Notably, PHF1-positive tau was detected in GFAP-positive astrocytes in the hippocampus of older mice, suggesting that astrocytes internalize and may contribute to tau spread (82) (Figure 1).

Heparan sulfate proteoglycans (HSPGs) are a well-conserved group of proteoglycans expressed on the cell surface of astrocytes and neurons (84, 85) that mediate targeted endocytosis (84), including that of purified prion proteins *in vitro* (86, 87). HSPGs were recently shown to interact with protein aggregates including α-synuclein, Aβ and tau (88–90). HSPGs regulate the uptake of synthetic tau fibrils (89) and human brain-derived tau (91) in human immortalized cell lines and mouse primary neuronal cultures. HSPGs vary in the length of their glycosaminoglycan chains and sulfation patterns, properties that are important for tau uptake in human embryonic kidney cells (92) and human iPSC derived neurons (93). Interestingly, tau fibrils are efficiently internalized in a HSPG-dependent manner by primary astrocytes exogenously expressing transcription factor EB (TFEB), a master regulator of lysosomal biogenesis (94). In contrast, monomeric tau appears to be taken up by astrocytes using an HSPG-independent mechanism (95). Together this suggests that multiple mechanisms are involved in tau uptake by astrocytes, that may be specific to tau aggregation state or conformation, as well as the HSPG profile of the cell type (96).

HSPGs can also partner with cell surface receptors to mediate the intake of protein aggregates. For example, HSPGs interact with members of the low-density lipoprotein receptor (LDLR) such as LRP1, to facilitate Aβ uptake and degradation by astrocytes (97, 98). Knockdown of LRP1 was recently shown to block the uptake of monomeric and oligomeric tau in a human neuroglioma cell line, and partially inhibit uptake of sonicated tau fibrils (99), warranting further investigation into how astrocytic LRP1 may mediate tau uptake and spread in tauopathies.

Astrocytes are an integral part of the glymphatic system of the brain, a clearance system of soluble proteins and solutes. The astrocytic water channel aquaporin-4 (AQP4), expressed at the astrocyte end feet, facilitates this process and is important for Aβ clearance (53, 100). Disruption to AQP4 may also contribute to tauopathy progression. In a mouse model of CTE, knockout of AQP4 exacerbated neurofibrillary tau pathology and neurodegeneration (101). Distinct phosphorylation marks in AQP4 have been reported in human post-mortem ARTAG samples relative to controls (102) that are suggested to increase water permeability of AQP4. However, the functional implications of these modifications in ARTAG remain to be explored (103, 104). A recent transcriptional analysis of cognitively-impaired subjects and controls showed that components of the dystrophin-associated complex, which anchors AQP4 at the perivascular astrocytic end foot, are associated with phosphorylated tau levels in the temporal cortex (54). This analysis also revealed other astrocyte endfoot candidate genes that significantly correlate with temporal cortex tau pathology. The authors speculate that endfoot functions of astrocytes may play a role in the accumulation of tau aggregates throughout the brain. Although AQP4 might contribute to the clearance of aberrant proteins early in the disease process, this function could become impaired at later stages, hindering the clearance of pathogenic tau.

## Tau Effects on Astrocyte Function

In addition to potential roles in tau spread, internalization of pathological forms of tau has been shown to disrupt a myriad of astrocytic functions, central for the maintenance and support of neurons. Oligomeric tau uptake alters calcium signaling and gliotransmitter release (e.g. ATP) *via* Ca^2+^-dependant mechanisms, to disrupt post-synaptic currents and downregulate pre- and post-synaptic markers in neuronal-astrocyte co-cultures (64), together suggesting that tau-induced changes to astrocyte function are toxic to neighboring neurons, at least *in vitro*. Astrocytes isolated from a transgenic tauopathy model (P301S) expressing a 4R mutant tau isoform also acquired early functional deficiencies that impaired their ability to support neurons in culture (105). Astrocytes from mouse models of tauopathies also show altered expression of neuronally regulated genes (106), indicating that the accumulation of abnormal tau species is sufficient to drive transcriptional and likely functional changes in astrocytes, *via* altered neuron-astrocyte interactions. In addition, human astrocytes differentiated from iPSCs harboring FTD-causing *MAPT* mutations display an increased vulnerability to oxidative stress and elevated protein ubiquitination, alongside disease-associated transcriptomic alterations (107).

The immune-related functions of astrocytes are a major contributor to neuroinflammatory response that directly alter neuronal integrity in neurodegenerative diseases (52). In particular, the complement cascade, which also involves microglia, has an important role in the accumulation of beta-amyloid pathology (108, 109). C3 is a major component of the complement cascade and is highly expressed in reactive astrocytes (71). C3, as well as its downstream receptor C3aR1, that is mainly expressed by microglia, (9), is upregulated in postmortem tauopathy brain and correlates with cognitive decline during disease progression (110). Levels of C3 also correlate with tau amounts in AD CSF (111). Ablation of C3aR or C3 in mouse models of tauopathy reversed neuronal loss and neurodegeneration (110, 111), alongside reduced numbers of GFAP-reactive hypertrophied astrocytes being apparent upon C3aR knockout (110). These data indicate that complement activation downstream of astrocyte reactivity may be an important driver of tauopathy.

Astrocytes, together with microglia, are also hypothesized to induce synaptic loss and neurotoxicity in tauopathies, as they do during development (112), through dysregulated synaptic pruning (113). Sleep deprivation is common in AD (114), where it is believed to be both a cause and consequence of neurodegenerative changes (114). Sleep deprivation leads to enhanced tau release and spread (115), alongside astrocyte-mediated synapse elimination (116). It is therefore possible that astrocyte engulfment of tau-containing synapses may be one route by which astrocytes contribute to tau spread in AD.

Ultimately, cross-talk between astrocytes and microglia forms part of a complex innate immune response that may be exacerbated during tauopathies in response to protein aggregates. Deeper investigation of these pathways may reveal novel targets that can be exploited to slow or halt disease progression.

## Discussion

Recent evidence has highlighted that altered astrocyte functions have detrimental consequences for neurons and may be a driver of neurodegenerative diseases. Astrocytes are closely associated with the accumulation of pathological forms of tau in tauopathies. There is some evidence that astrocytes internalize tau aggregates, *via* mechanisms that are not yet fully understood, and contribute to tau pathology spread across the brain and tau aggregate clearance *via* the glymphatic system. However, astrocytes show significant regional heterogeneity and more work is needed to better understand the contribution of different astrocyte subtypes in affected brain regions at different disease stages. Such understanding may aid in the development of astrocyte-targeted therapies for tauopathies. Astrocyte-targeted therapeutic approaches have been well-described elsewhere including by Sadik and Liddelow (70), and could include antagonists that prevent tau uptake by astrocytes to reduce tau spread, agents that prevent the release of neurotoxic astrocyte secretions or their uptake by neurons, or therapies that restore physiological astrocyte functions including their trophic support for neurons and synapses, maintenance of the blood brain barrier, and roles in the glymphatic clearance of protein aggregates.

## Author Contributions

MR, PB-L, LJ, BP-N, and WN wrote and edited the manuscript. All authors contributed to the article and approved the submitted version.

## Conflict of Interest

The authors declare that the research was conducted in the absence of any commercial or financial relationships that could be construed as a potential conflict of interest.
